# Nuclear Modifier *MTO2* Modulates the Aminoglycoside-Sensitivity of Mitochondrial 15S rRNA C1477G Mutation in *Saccharomyces cerevisiae*

**DOI:** 10.1371/journal.pone.0081490

**Published:** 2013-12-10

**Authors:** Xiangyu He, Xiaoyu Zhu, Xuexiang Wang, Wei Wang, Yu Dai, Qingfeng Yan

**Affiliations:** College of Life Science, Zhejiang University, Hangzhou, Zhejiang, China; CNR, Italy

## Abstract

The phenotypic manifestations of mitochondrial DNA (mtDNA) mutations are modulated by mitochondrial DNA haplotypes, nuclear modifier genes and environmental factors. The yeast mitochondrial 15S rRNA C1477G (P^R^ or P^R^_454_) mutation corresponds to the human 12S rRNA C1494T and A1555G mutations, which are well known as primary factors for aminoglycoside-induced nonsyndromic deafness. Here we report that the deletion of the nuclear modifier gene *MTO2* suppressed the aminoglycoside-sensitivity of mitochondrial 15S rRNA C1477G mutation in *Saccharomyces cerevisiae*. First, the strain with a single mtDNA C1477G mutation exhibited hypersensitivity to neomycin. Functional assays indicated that the steady-state transcription level of mitochondrial DNA, the mitochondrial respiratory rate, and the membrane potential decreased significantly after neomycin treatment. The impaired mitochondria could not produce sufficient energy to maintain cell viability. Second, when the *mto2* null and the mitochondrial C1477G mutations co-existed (*mto2*(P^R^)), the oxygen consumption rate in the double mutant decreased markedly compared to that of the control strains (*MTO2*(P^S^), *mto2*(P^S^) and *MTO2*(P^R^)). The expression levels of the key glycolytic genes *HXK2*, *PFK1* and *PYK1* in the *mto2*(P^R^) strain were stimulated by neomycin and up-regulated by 89%, 112% and 55%, respectively. The enhanced glycolysis compensated for the respiratory energy deficits, and could be inhibited by the glycolytic enzyme inhibitor. Our findings in yeast will provide a new insight into the pathogenesis of human deafness.

## Introduction

The phenotypic manifestations of mitochondrial DNA (mtDNA) mutations are modulated by mitochondrial DNA haplotypes, nuclear modifier genes and environmental factors [1], [2]. A typical example is the human mtDNA 12S rRNA A1555G mutation, which is well known as a primary determinant of aminoglycoside-induced nonsyndromic deafness [3], [4], [5]. However, individuals carrying the A1555G mutation exhibit diverse clinical phenotypes ranging from normal hearing to severe deafness. This suggests that the clinical symptom may be also under the regulation of nuclear genes and environmental factors [3], [5], [6], [7]. So far, most studies on the mitochondrial A1555G mutation have had their main focus upon one factor, or the interaction between two factors. The combined effect of mtDNA mutation and nuclear modifier genes have been presented in several studies where environmental influences appear to play a role but remain poorly understood [7], [8].

**Figure 1:**
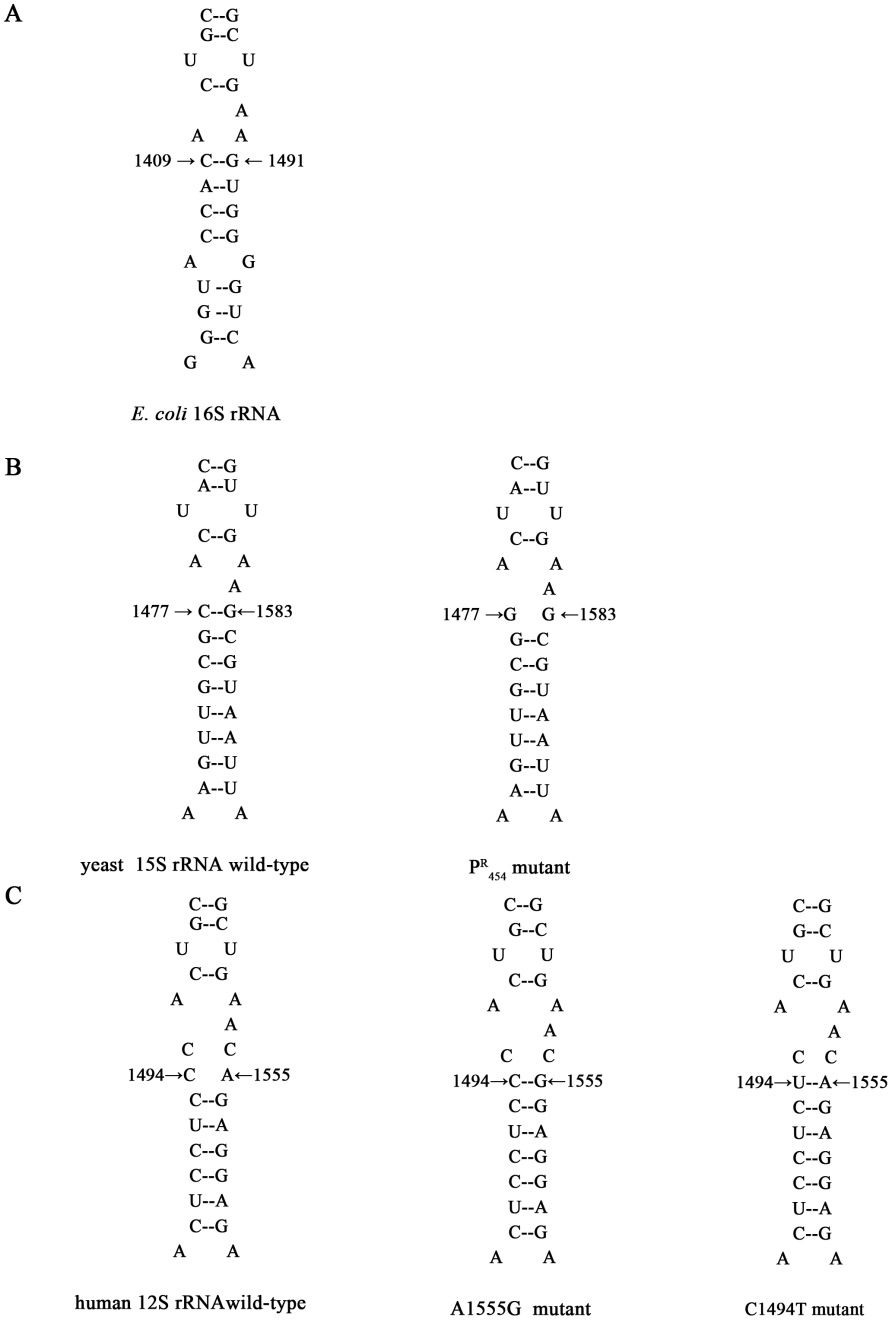
Secondary structure of small rRNA decoding sites in yeast and human mitochondria. ***A***, secondary structure of *E. coli* small ribosome rRNA decoding site. ***B***, wild type and P^R^ mutant forms of yeast 15S rRNA decoding sites, and the base-pair affected by P^R^ mutation are indicated by arrowheads. ***C***, the corresponding regions of human mitochondrial 12S rRNA are shown as the wild type version and versions containing A1555G and C1494T mutations, respectively.

The yeast mitochondrial 15S rRNA C1477G mutation corresponds to the human 12S rRNA C1494T and A1555G mutations (Figure 1). Yeast carrying this mutation is often used as a genetic model to investigate nuclear-mitochondrial interactions [8], [9], [10]. However, in a contrasting case to that of A1555G mutation in humans, the exact impact of the C1477G mutation on yeast antibiotic sensitivity remains disputed. Kutzleb *et al* first reported a yeast strain resistant to paromomycin, and later Li *et al* identified a C1477G mutation in the 3′ end of mitochondrial 15S rRNA gene from the same strain [11], [12]. This mutation locates at a highly conserved rRNA decoding site (A-site), and alters the base pairing between C1477 and G1583 nucleotides. However, Weiss-Brummer *et al* did not observe the paromomycin resistant phenotype when culturing the C1477G mutant strains on paromomycin-containing medium [13]. The human A1555G mutation generates comparable modifications of secondary structure to those of the yeast C1477G mutation (Figure 1), and makes individuals highly susceptible to aminoglycosides. Aminoglycosides are widely used clinically, but their ototoxiticy limits the range of their application. The binding of aminoglycoside antibiotics to mitochondrial small ribosomal RNA carrying the A1555G or C1494T mutations leads to a mitochondrial respiratory defect and eventually to drug-induced deafness [3], [14], [15]. Thus, the penetrance and expressivity of the A1555G mutation in carriers are often enhanced by aminoglycosides.

In *Saccharomyces cerevisiae*, the mitochondrial 15S rRNA C1477G mutation combining with the nuclear gene *mss1, mto1* or *mto2* null mutations generated a respiratory deficient phenotype. In yeast mitochondria, Mto2p, along with Mto1p and Mss1p, participates in the same pathway catalyzing the formation of the hypermodified base 5-mthyl-aminomethyl-2-thio-uridine (mnm^5^s^2^U34) in the wobble position of tRNA^Lys^, tRNA^Glu^ and tRNA^Gln^ [16], [17], [18]. *MTO2* encodes a mitochondrial tRNA-specific 2-thiouridylase, which is responsible for 2-thiolation of the U34 nucleotide in the tRNA anti-codon loop. This is the initial step of the mnm^5^s^2^U34 modification pathway [17]. This kind of modification is crucial to the mitochondrial translational fidelity and the efficiency of protein synthesis, because the modified uridine base increases both the stability of these tRNAs and the capacity of codon recognition at the ribosomal A-site. Meanwhile, *TRMU* (human *MTO2*) was the first identified nuclear modifier gene regulating the phenotypic manifestation of the mitochondrial A1555G mutation [19]. These studies indicated a complex interaction between the *MTO2* (*TRMU*) gene and the mitochondrial small rRNA.

The aim of the present study is to determine the interactions among mitochondrial rRNA mutations, nuclear genes, and environmental factors, as well as their combined effects on cellular function. For the first time, we found that the nuclear gene *MTO2* could modulate aminoglycoside antibiotic sensitivity induced by the mitochondrial C1477G mutation. Yeast carrying the 15S rRNA C1477G mutation alone exhibited hypersensitivity to aminoglycosides, while *MTO2* deletion suppressed aminoglycoside-sensitivity in yeast carrying the mitochondrial C1477G mutation. The underlying molecular mechanism was also analyzed. Biochemical activities of cells and mitochondria, including those of respiratory rate, mitochondrial membrane potential, and expression levels of mitochondrial and glycolytic genes, were determined.

## Materials and Methods

### Yeast Strains and Culture Conditions

The genotypes and sources of yeast *S. cerevisiae* strains had been described elsewhere [8], [9]. All yeast strains were cultured in YPD complete medium (1% yeast extract, 1% peptone and 2% glucose). Aminoglycoside antibiotic-containing media were prepared by adding 100 mg/ml stock solutions of antibiotics into the YPD medium after sterilization. The final working concentration of each antibiotic was determined according to the data from a minimal inhibitory concentration assay. 100 mM 2-deoxy-glucose (2-DG) stock solution was prepared in DMSO, and the final concentration of 2-DG in YPD was 2.5 mM.

### Minimal Inhibitory Concentration (MIC) Assay

The MIC_90_ was defined as the minimal antibiotic concentration at which 90% of organism growth was inhibited by the drug after incubation for 24 hours at 30°C. Neomycin and paromomycin were purchased from Sigma. To determine the MIC_90_ of each aminoglycoside antibiotic on each yeast strain, cultures were grown to a mid-log phase at 30°C in YPD medium. Then the cells were harvested and subcultured in a YPD medium containing 2 fold gradient dilutions of antibiotics, where the starting cell density was 2×10^5^ cells/ml [20], [21].

### Phenotypic Analysis and Growth Curve

For the phenotypic analysis, yeast strains were inoculated and grown in a liquid YPD medium at 30°C overnight until they reached the exponential growth phase. The cultures were then harvested, serially diluted, and spot on the YPD medium and YPD containing aminoglycosides, respectively. The plates were incubated at 30°C for 3 days and photographs were taken [8].

For the growth curve analysis, strains were cultured in liquid YPD both in the absence and presence of aminoglycoside with a starting cell density of 0.01 OD_600_. The growth conditions were monitored by measuring the OD_600_ every two hours for the first 20 hours [22].

### Oxygen Consumption Assay

Oxygen consumption rate (OCR) measurements were carried out as described elsewhere with minor modifications [23]. Yeast cells were cultured in liquid YPD medium at 30°C overnight. Then cells were harvested and subcultured at the starting density of 2×10^5^ cells/ml in both antibiotic-containing and antibiotic-free media for 16 hours. After validation of the phenotypes, cells were harvested in the mid log-phase and subsequently seeded in pre-coated Poly-D Lysine (50 μg/ml) XF 96-well microplates (Seahorse Bioscience) at 4×10^5^ cells per well, then spun down, and inoculated at 30°C for 30 min. Subsequently the oxygen consumption rate was measured according to manufacturer’s instructions on a Seahorse XF96 Extracellular Flux Analyzer. The OCR was shown as picomole oxygen per minute per cell (pmol/min/cell).

### Mitochondrial Membrane Potential Assay

For the mitochondrial membrane potential assay, yeast cells were harvested from both antibiotic-free and antibiotic-containing media. 2×10^6^ cells were resuspended in 1 ml supernatant and incubated with Rhodamine 123 (5 μg/ml) for 20 min at 30°C in a shaker. The cells were then centrifuged and washed with a phosphatebuffered saline (PBS) 3 times. Cell pellets were resuspended in 20 μl PBS and visualized with a Carl Zeiss 710 LSM microscope [24].

### Northern Blot Analysis

Total cellular RNA was obtained from yeast cultures (2.0×10^7^ cells) using TRIzol Reagent (Life Technologies) according to the manufacturer’s instructions. Equal amounts (10 μg) of total RNA were separated by electrophoresis through a 1.5% formaldehyde denaturing agarose gel, transferred onto a positively charged nylon membrane (Amersham) and hybridized with a DIG-labeled ATP6-specific antisense RNA probe. The blot was then stripped with stripping buffer (50% formamide, 50 mM Tris/HCl, pH 7.5, 5% SDS) and hybridized with the *COX1*, *CYTB*, *ATP9*, *15S rRNA* and *21S rRNA* probes, respectively. Finally, the blot was hybridized with a nuclear encoded *25S rRNA* probe as an internal control. For the transcriptional assay of glycolytic genes, probes specific to *HXK2*, *PFK1* and *PYK1* were used in a northern blot, and *25S rRNA* was hybridized as an internal control.

### Hexokinase Protein Expression Assay

Antibodies were purchased from Santa Cruz Biotechnologies (Santa Cruz, CA, U.S.A.). Tubulin was used as an internal control. The strains were cultured for 16 hours in YPD at 30°C with or without antibiotic, before processing. Protein samples were prepared, and 50 µg of total protein for each sample was loaded and SDS-PAGE was performed as described elsewhere, followed by transfer to a PVDF membrane [25]. Immunoblotting was performed as previously described with the secondary antibody goat anti-rabbit (or rabbit anti-goat) IgG conjugated with horseradish peroxidase, followed by development with ECL solution (Santa Cruz).

### Statistical Analysis

All experiments were repeated at least three times and the representative data were presented as means±SD. One-way analysis of variance (ANOVA) was performed to determine the significance between groups. *P*<0.05 was considered as statistically significant: *(*P*<0.05), **(*P*<0.01).

## Results

### Growth Activities of Yeast Strains in the Presence of Neomycin

Our previous studies had revealed that the mitochondrial 15S rRNA C1477G mutation combined with *mto2* null mutation impaired mitochondrial function in yeast [8]. Here we have further determined the effects of aminoglycoside antibiotics and the interrelationships among nuclear modifier gene, mitochondrial rRNA mutation and antibiotics. To examine the functional consequence of antibiotics on yeast, yeast cells carrying mitochondrial 15S rRNA C1477G and/or nuclear *mto2* null mutation were cultured in YPD medium containing neomycin. Cellular growth activities were then measured. Yeast of wild type 15S rRNA and *MTO2* genotypes were used as positive controls.

**Figure 2:**
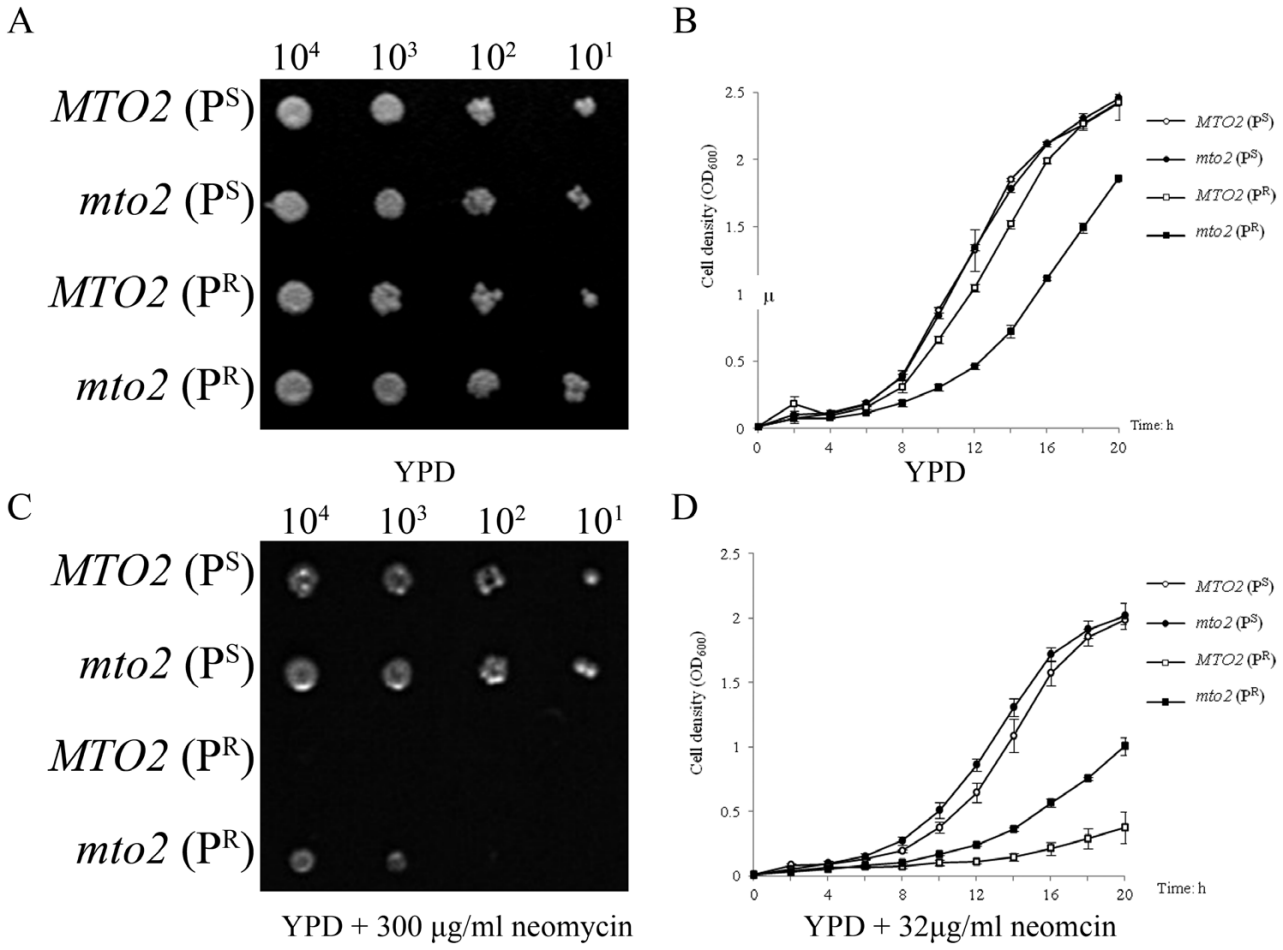
Growth activities of different yeast strains. ***A***, Series dilutions of each strain were spotted onto a 2% glucose medium (YPD) and the plate was incubated at 30°C for 72 hours. ***B***, Growth curves analysis of yeast strains in the absence of neomycin in 20 hours. ***C***, Growth activities of each strain when grown on medium containing neomycin after 72 hours incubation. ***D***, Growth curves of strains cultured in YPD containing 32 µg/ml neomycin.

The phenotypes of yeast with different genetic backgrounds indicated that a mutation in mitochondrial 15S rRNA leads to neomycin sensitivity. All four strains grew well on the YPD medium and no difference in growth was observed (Figure 2A). However, when grown on medium containing 300 μg/ml neomycin, the growth of the strains carrying mitochondrial 15S rRNA mutations were inhibited compared to wild type P^S^ strains (Figure 2C). These phenotypes were consistent with yeast growth curves in liquid media (Figure 2B, D). When cultured in media containing neomycin, the two mitochondrial mutant strains exhibited delayed logarithmic phases, while the P^S^ strains entered rapidly into logarithmic growth at approximately 6 hours after inoculation.

Although the deletion of the nuclear gene *MTO2* had no effect on P^S^ strains, it significantly altered neomycin susceptibility for strains carrying a mitochondrial 15S rRNA mutation. The *MTO2*(P^R^) strain was totally inhibited by neomycin after 3 days’ incubation (Figure 2C). However, the double mutant *mto2*(P^R^) displayed a much better growth activity and was only partially inhibited. Nevertheless, in the aspect of growth curves, the two 15S rRNA mutant strains presented quite different log-phases. The cell density of *MTO2*(P^R^) was 0.3 OD_600_ after 20 hours’ incubation in the neomycin medium, while *mto2*(P^R^) reached a final cell density of 1.01 OD_600_. These data strongly indicates that the nuclear gene *MTO2* regulates aminoglycoside sensitivity in yeast carrying the mitochondrial 15S rRNA C1477G mutation, and that deletion of *MTO2* suppresses this sensitivity.

### Mitochondrial Respiratory Rates

**Figure 3:**
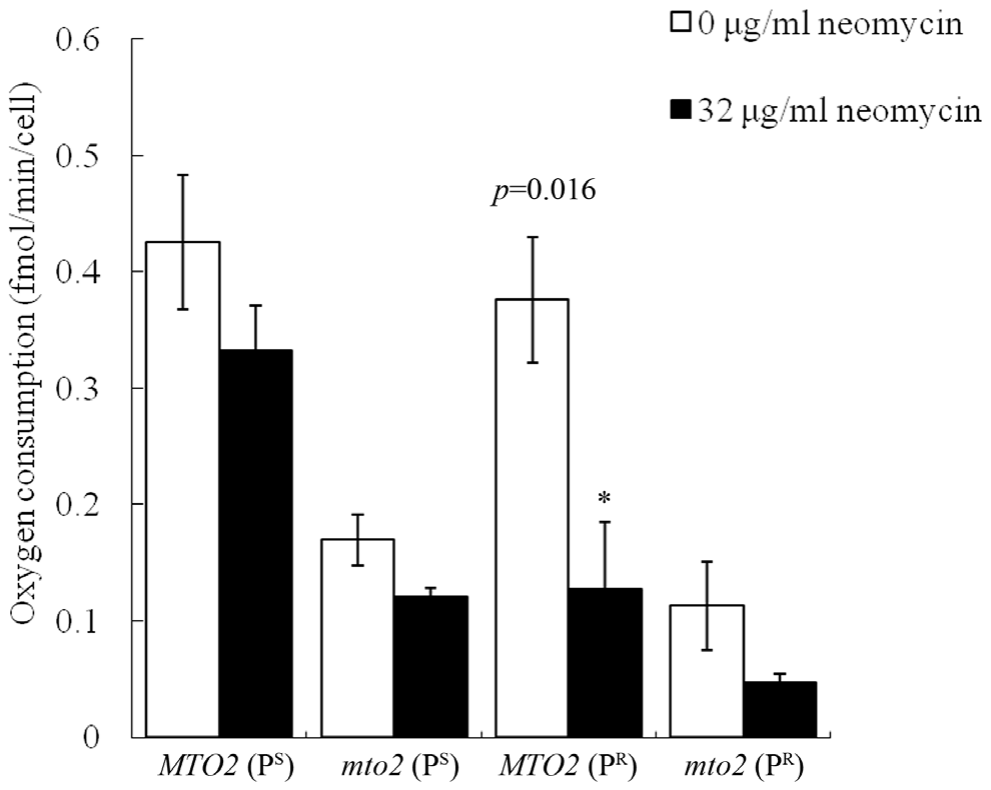
Assay of Oxygen consumption rates. Cells were harvested in the mid log-phase. The oxygen consumption rate of each yeast strain was measured at the density of 4×10^5^ cells/well by FX-96 oxygraph (SeaHorse Biosciences) in the absence or presence of 32 μg/ml neomycin. The results are shown as means±SD of triplicate.

To determine the functional impacts of neomycin on yeast mitochondria *in vivo*, we examined the respiratory rates of the strains by measuring the rate of oxygen consumption. In the absence of neomycin, the wild-type strain *MTO2*(P^S^) had a basal respiratory rate of 0.43 fmol/min/cell (Figure 3). Mitochondrial function was slightly disturbed by the mitochondrial 15S rRNA mutation, and the basal respiratory rate of *MTO2*(P^R^) was 11.63% lower than that of *MTO2*(P^S^). Deletion of the *MTO2* gene had a significant influence on mitochondrial respiration under both P^S^ and P^R^ mitochondrial genetic backgrounds, and 60.47% (*p* = 0.0203) and 71.05% (*p* = 0.01222) declines were observed in *mto2* null strains, respectively.

We further analyzed the respiratory rates of yeast in the presence of neomycin. The respiratory rates of strains carrying the mitochondrial 15S rRNA mutation were significantly inhibited by neomycin as compared to the control group. After neomycin treatment, the respiratory rate of *MTO2*(P^R^) and the double mutant *mto2*(P^R^) declined by 65.79% and 58.33%, respectively. However, in the P^S^ strains, only 23.26% and 29.41% declines were observed, respectively, suggesting that the wild type mitochondria were much less sensitive to neomycin. These data indicated that neomycin was more toxic to yeast cells carrying the mitochondrial 15S rRNA mutation.

### Mitochondrial Membrane Potential Assay

**Figure 4:**
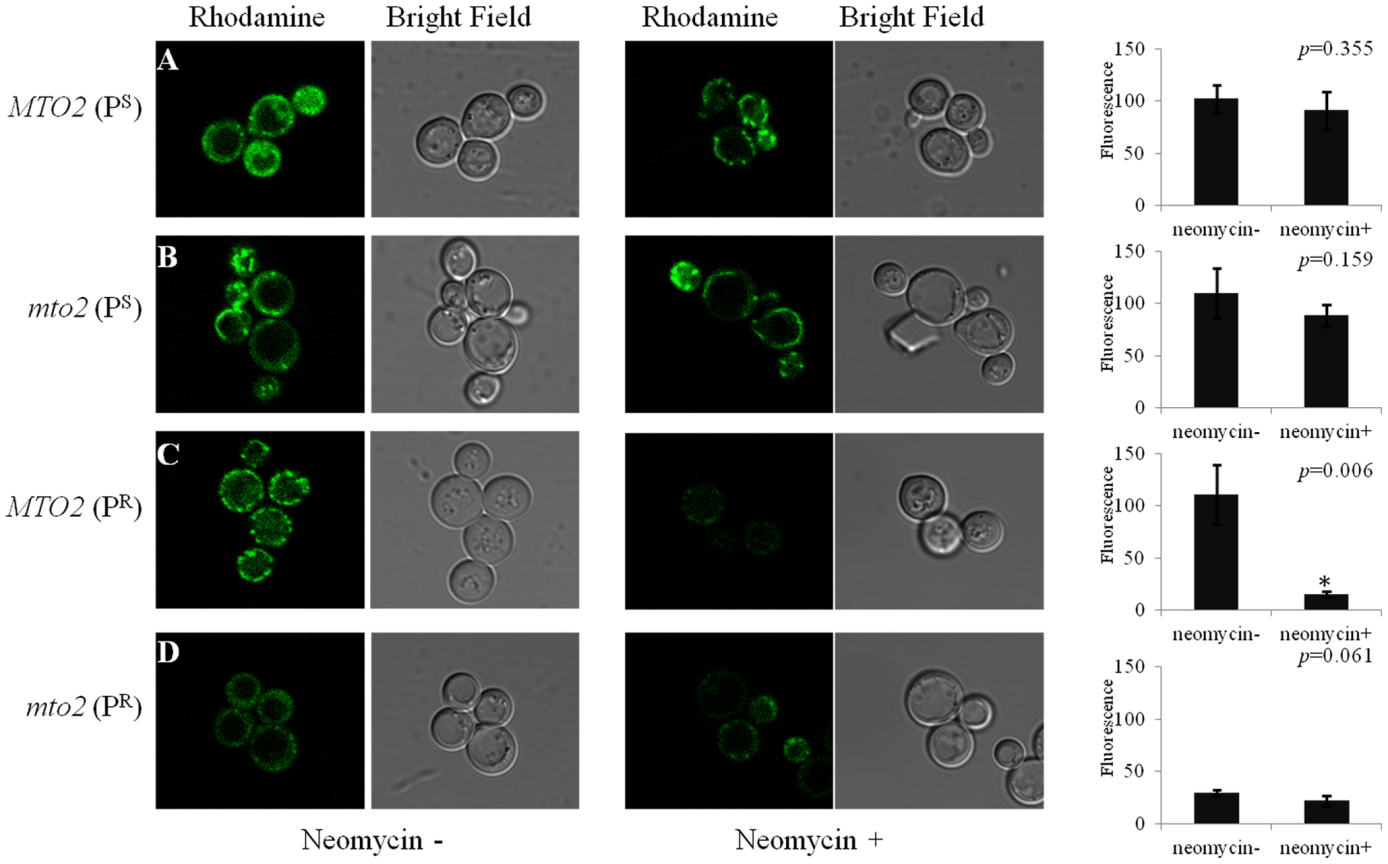
*In vivo* staining of the mitochondrion to measure the membrane potential by Rhodamine 123 dyes. 2×10^6^ cells in 1 ml supernatant were incubated with Rhodamine 123 (5 µg/ml) for 20 min at 30°C. Cell pellets were resuspended in 20 µl PBS and visualized with Carl Zeiss 710 LSM microscopy. The relative fluorescence signal of each strain is shown in the right panel.

The rhodamine 123 fluorescent dye was used as an indicator of yeast mitochondrial membrane potential as the uptake of the dye was dependent on the mitochondrial inner membrane potential [24]. The fluorescent signal strength was altered by different genetic and antibiotic factors (Figure 4). In the control group without neomycin, *MTO2*(P^S^), *mto2*(P^S^) and *MTO2*(P^R^) all had relatively high basal membrane potentials. However, the double mutant *mto2*(P^R^) strain had a much lower membrane potential, equivalent to 29% of that of *MTO2*(P^S^). This indicates that the *mto2* null mutation and mitochondrial 15S rRNA point mutation have synergistic suppressive effects on mitochondrial function.

When cultured in media containing neomycin, the mitochondrial membrane potentials in yeast cells were inhibited to different extents. The membrane potentials of the two strains with a wild type mitochondrial genetic background declined by 11% and 19% after neomycin treatment, respectively. This suggests that neomycin has only a slight effect on wild type mitochondria. In contrast, the membrane potential of *MTO2*(P^R^) was significantly decreased by 86%, and the green fluorescent signal was almost invisible compared to the untreated control. Moreover, the mitochondrial membrane potential in the *mto2*(P^R^) strain was further attenuated by neomycin, and in a manner somewhat parallel to the change of its respiratory rate.

### Mitochondrial DNA Transcription Assay

**Figure 5:**
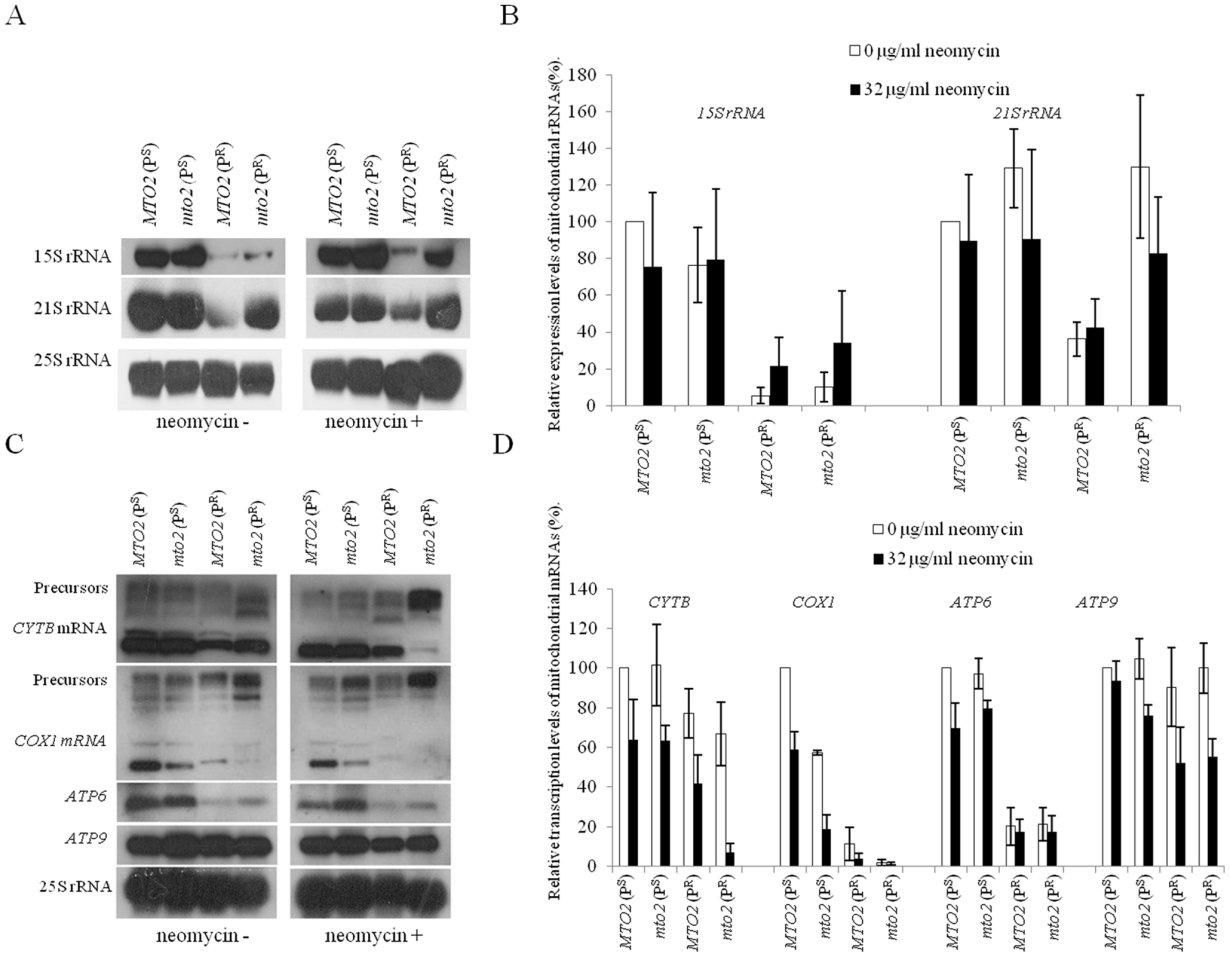
Northern blot analysis of transcription levels of mitochondrial genes. ***A***, northern blot analysis of mitochondrial *15S rRNA* and *21S rRNA*. The nuclear encoded *25S rRNA* was hybridized as a internal control. ***B***, quantitative analysis of mitochondrial rRNA transcription levels, where each calculation was based on three independent determinations of each RNA in each yeast strain. ***C***, northern blot analysis of *CYTB*, *COX1*, *ATP6* and *ATP9*. ***D***, quantitative analysis of mitochondrial mRNA transcription levels.

The mitochondrial 15S rRNA C1477G mutation affected steady levels of mitochondrial rRNAs. In the control group, *MTO2*(P^R^) and *mto2*(P^R^) had significantly lower 15S rRNA transcripts than wild type P^S^ strains (Figure 5A). This suggested that the C1477G mutation had impaired the stability of 15S rRNA. However, the expression patterns of *21S rRNA* between P^R^ strains were quite different, suggesting that the large subunit and small subunit of mitochondrial ribosomes were differentially affected by the C1477G mutation. Unexpectedly, neomycin had no significant inhibitory effect on 15S rRNA and 21S rRNA in the P^R^ strains.

Previous studies have revealed that the *mto2* null mutation has effects on the expression of mitochondrial genes and the maturation of *COX1* and *CYTB* primary transcripts [8], [26]. We further performed transcription assays in the presence of neomycin. The mitochondrial 15S rRNA C1477G mutation affected the expression levels of *COX1* and *ATP6*, but *CYTB* and *ATP9* were less affected (Figure 5C). This result suggests that the transcription of mitochondrial genes in yeast may be differentially regulated. A novel phenomenon was observed that neomycin significantly affected precursor maturation of *COX1* and *CYTB* in the *mto2*(P^R^) strain. However, the accumulation of precursors in the *MTO2*(P^R^) was less observed. This may indicate that *MTO2* interacts functionally with the mitochondrial RNA processing machinery.

### Expression of Regulatory Genes in Glycolytic Pathway

**Figure 6:**
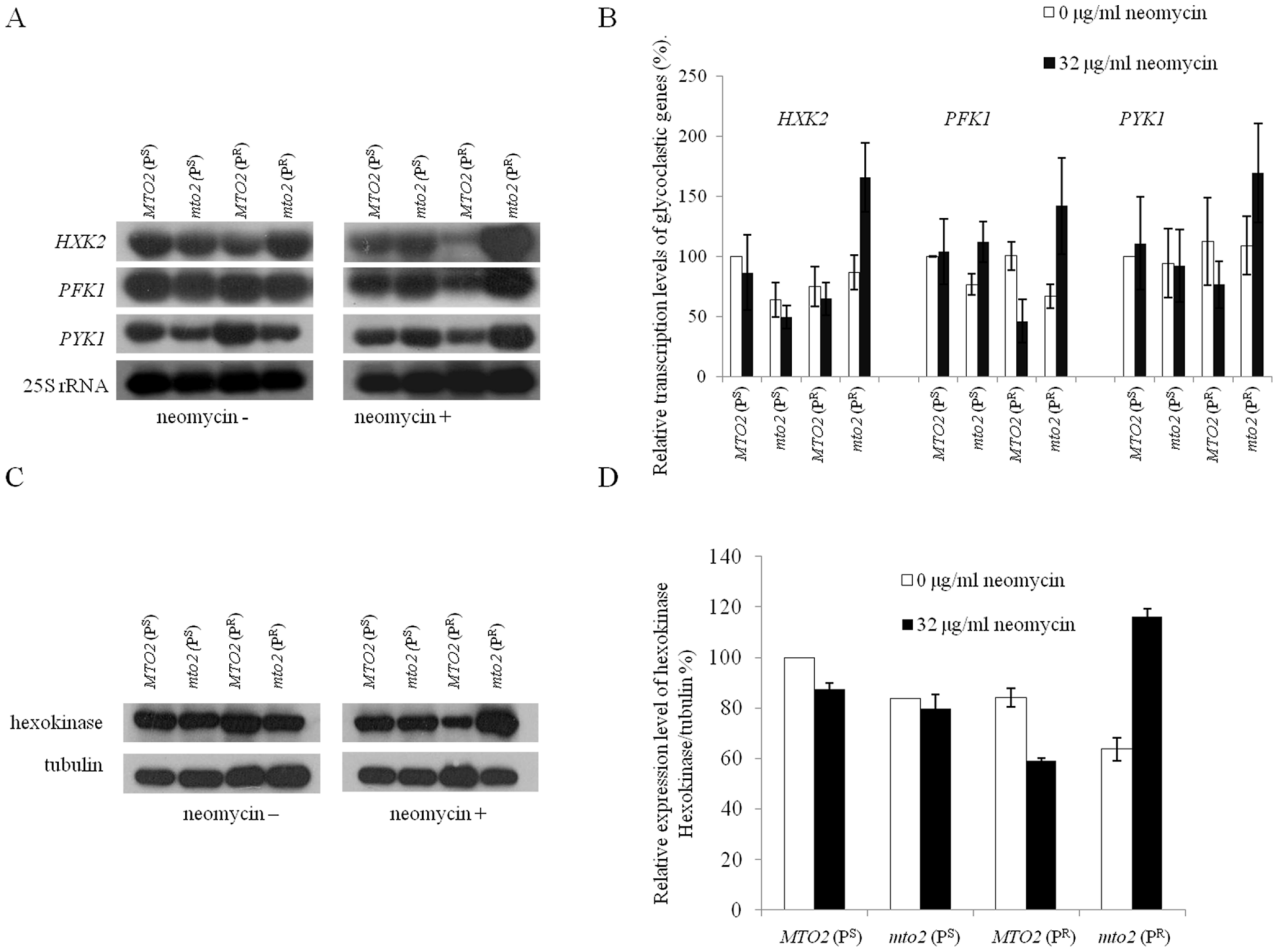
Steady-state level of key glycolytic genes. ***A***, northern blot analysis of *HXK2*, *PFK1* and *PYK1* transcription levels. The nuclear encoded 25S rRNA was hybridized as an internal control. ***B***, relative expression levels of these genes were calculated by three determinations. ***C***, translational levels of hexokinase in each strain, tubulin was as an internal control. ***D***, calculation of the relative expression level of hexokinase.

Hexokinase (*HXK*), phosphofructokinase (*PFK*) and pyruvate kinase (*PYK*) are key regulators in the glycolytic pathway, where *PFK* and *PYK* are enzymes involved exclusively in glycolysis [27]. Steady-state levels of *HXK2*, *PFK1* and *PYK1* mRNAs were analyzed by northern blot using DIG-labeled anti-sense RNA probes. The blot was then stripped and re-hybridized with a *25S rRNA* probe as an internal control. In the absence of neomycin, the transcription levels of glycolytic genes differed slightly (Figure 6A). The *MTO2*(P^R^) strain had a lower *HXK2* mRNA level, but the *PFK1* and *PYK1* transcription levels were higher than those in other three strains. However, after neomycin treatment, all of the three genes in *MTO2*(P^R^) were further down regulated. In *mto2*(P^R^) strains, *HXK2*, *PFK1* and *PYK1* transcription levels were simultaneously stimulated by neomycin and up-regulated by 89%, 112% and 55%, respectively. In contrast, transcription levels of these three genes in *MTO2*(P^R^) were further impaired by 14%, 54% and 32%, respectively.

The expression level of hexokinase was also analyzed by western blotting with an anti-hexokinase antibody. Hexokinase catalyzes the initial step of glycolysis, which irreversibly converts glucose to glucose-6-phosphate, a step which also plays an important regulatory role in glycolysis [28]. When cultured in media without neomycin, the hexokinase levels in *mto2*(P^S^), *MTO2*(P^R^) and *mto2*(P^R^) strains were 83%, 84% and 64% compared to wild type *MTO2*(P^S^) (Figure 6C). After neomycin treatment, only the double mutant *mto2*(P^R^) had an increased hexokinase level, which was 82% higher than its control. However, the other three strains had 12%, 5%, and 30% declines compared to the non-treated controls. These data indicated that the glycolytic pathway in the *mto2*(P^R^) strain was up-regulated after neomycin treatment. In summary, we concluded that the *MTO2*(P^R^) strain was significantly inhibited by neomycin because both aerobic and anaerobic metabolisms were suppressed. The up-regulated glycolysis in the *mto2*(P^R^) strain may compensate for mitochondrial dysfunction induced by neomycin and thus this strain was less sensitive to neomycin.

### Effects of Glycolytic Inhibitor on Growth Activities of Yeast Strains

**Figure 7:**
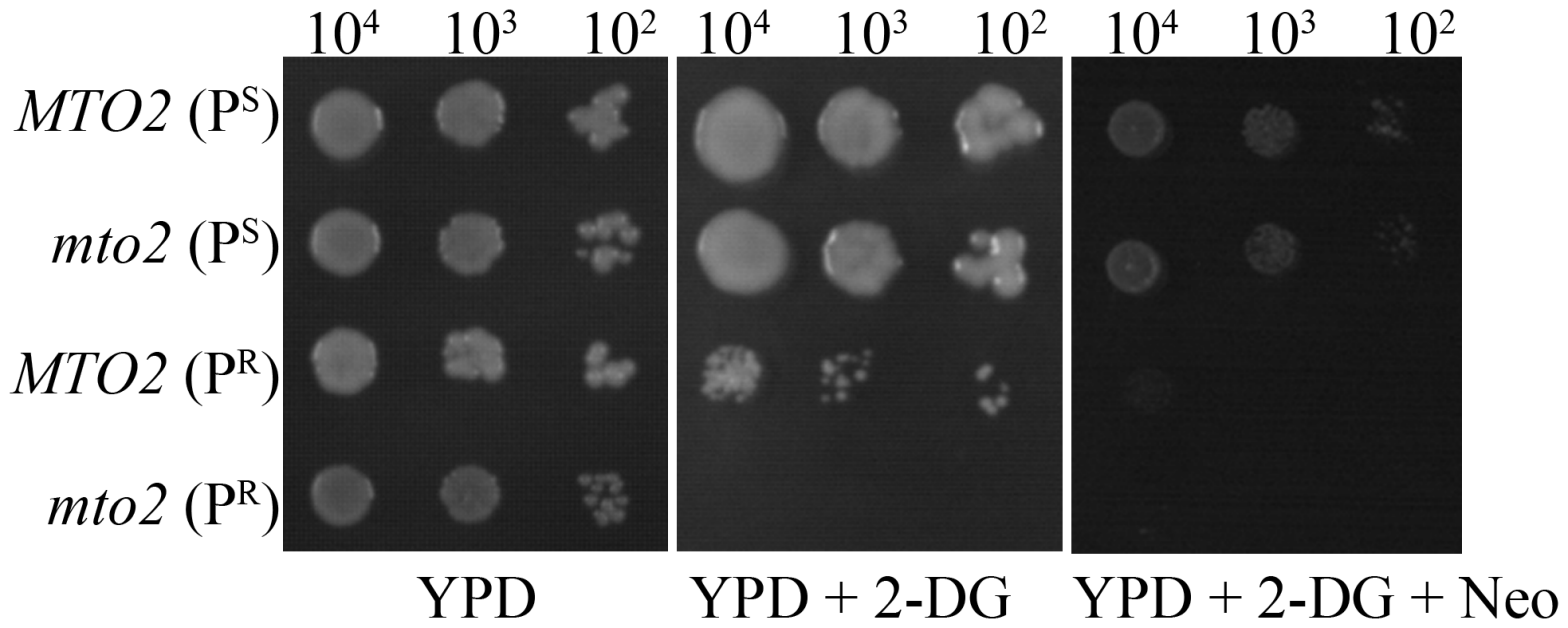
Properties of yeast cells on YPD plate in the presence of glycolytic inhibitor 2-Deoxy-D-glucose (2-DG). Series dilutions of each strain were spotted on medium and the plate was incubated at 30°C for 72 hours. ***A***, on YPD medium. ***B***, on YPD medium supplemented with 2.5 mM 2-DG. ***C***, on YPD medium supplemented with 2.5 mM 2-DG and 300 µg/ml neomyocin.

2-DG, as a glucose analogue, competitively inhibits hexokinase and blocks the glycolytic pathway [29]. On a YPD medium supplemented with 2.5 mM 2-DG, the double mutant *mto2*(P^R^) strain cannot grow because of its dependence on the glycolytic metabolism, but *MTO2*(P^S^), *mto2*(P^S^) and *MTO2*(P^R^) strains can maintain their growth through mitochondrial respiration (Figure 7). On a YPD medium supplemented with 2.5 mM 2-DG and 300 μg/ml neomyocin, the *mto2*(P^R^) strain exhibits slight growth (Figure 7). These data were consistent with the assays of oxygen consumption rates and steady-state level of glycolytic genes.

## Discussion

In this study, we analyzed the interactions among the mitochondrial 15S rRNA C1477G mutation, the nuclear modifier gene *MTO2*, and neomycin, as well as their combined effects on mitochondrial function. The results suggest that yeast cells carrying the C1477G mutation in the mitochondrial 15S rRNA gene exhibit hypersensitivity to neomycin, while deletion of *MTO2* gene suppresses this sensitivity. Kutzleb first described paromomycin-resistance (P^R^) as a consequence of 15S rRNA C1477G mutation, because yeast with this mutation could survive on 2 mg/ml paromomycin-containing medium [12]. In contrast, Weiss-Brummer and colleagues reported that the C1477G mutation alone was not sufficient to form a paromomycin-resistant phenotpye [12], [13]. Our results supported Weiss-Brummer’s study. When cultured in a YPD medium containing a low dosage of neomycin, yeast cells with the mitochondrial C1477G mutation were significantly inhibited compared to those of the wild type strain. Moreover, P^R^ strains were also sensitive to other aminoglycosides, such as paromomycin and ribostamycin (Figure S1). The MIC_90_ values of the neomycin of the wild type P^S^ strains were both 128 μg/ml, while the *MTO2*(P^R^) and *mto2*(P^R^) had the MIC_90_ values of 32 μg/ml and 64 μg/ml, respectively (Table S1). A spot assay displayed a totally inhibited phenotype of *MTO2*(P^R^) by neomycin, while the wild type *MTO2*(P^S^) remained unaffected. Functional assays revealed that both the mitochondrial respiratory rate and membrane potential were aggravated after neomycin treatment. Meanwhile, the transcription levels of mitochondrial genes in C1477G mutant strains were sharply attenuated compared to those of the 15S rRNA wild type strains. Neomycin and other aminoglycosides are known to exert antibacterial effects by disrupting the elongation step in protein synthesis with ribosomes [30]. These antibiotics interfere with translation by inhibiting the translocation of the tRNA-mRNA complex from the ribosomal A site to the P site [31]. Several mutations in eukaryotic mitochondrial rRNA genes, such as those of human A1555G and C1494T mutations, alter the binding activity of aminoglycosides to ribosomes [3], [15]. The binding of antibiotics to mutant rRNA may further interfere with the movement of the tRNA-mRNA complex on the ribosome [32]. The yeast mitochondrial C1477G mutation is well known to form an identical secondary structure to that of human A1555G and C1477G mutations, and the structure of A1555G and C1494T mutant forms in humans is highly susceptible to neomycin binding [14]. Therefore, the yeast mitochondrial ribosome with the C1477G mutation may exhibit a comparable affinity to neomycin [8], [9].

Another interesting finding is that the nuclear modifier gene *MTO2* regulates the antibiotic sensitivity of yeast carrying the C1477G mutation. The MIC_90_ value of neomycin in *mto2*(P^R^) was elevated 2-fold compared to that in *MTO2*(P^R^), suggesting that the *mto2* null mutant was less sensitive to neomycin in the mitochondrial C1477G mutation. The growth curves clearly indicated that the *mto2*(P^R^) strain had a more vigorous growth activity than *MTO2*(P^R^) in the presence of neomycin. We also studied the neomycin intake and the expression levels of neomycin-resistant genes in these strains. No difference was observed (data unpublished). Expression assays of genes involved in the glycolytic pathway displayed distinct patterns between the two C1477G mutant strains when treated with neomycin. The transcription levels of *HXK2*, *PFK1* and *PYK1* in *MTO2*(P^R^) strains were further suppressed after neomycin exposure, while in the *mto2*(P^R^) strain these genes were up-regulated as compared to neomycin-free controls. This result indicates that, although deletion of the *MTO2* gene reduces oxidative phosphorylation in the presence of a C1477G mutation, it might also activate the glycolytic metabolic pathway and make the strain more dependent on glycolysis for its energy supply. Thus, the double mutant strain *mto2*(P^R^) exhibits a partially restored phenotype in a neomycin-containing medium. We also confirmed that the up-regulated glycolytic pathway in *mto2*(P^R^) cells could be suppressed by the hexokinase inhibitor 2-DG. This suppression again made the *mto2*(P^R^) strain sensitive to neomycin.

*MTO2* is highly conserved evolutionally from *E.coli* to humans, and in all eukaryotes this gene encodes a mitochondrial-specific tRNA 2-thiouridylase, catalyzing the formation of cmnm^5^s^2^U34 in mitochondrial tRNA^Glu^, tRNA^Gln^ and tRNA^Lys^ [17], [33]. According to our data, we suspect that the human homolog of *MTO2* may also regulate the aminoglycoside sensitivity of the A1555G mutation. Indeed, according to some epidemiological studies, the aminoglycoside sensitivity of A1555G mutation carriers varies between different ethnic populations where Asian people, for example, developed deafness more rapidly and severely after exposure to aminoglycosides. This could be associated with different nuclear genetic background, such as those of the various *TRMU* genotypes. Recently, a series of reports regarding *TRMU* mutation has been published, and it seems that these mutations are much more common in European and Latin American populations [19], [34], [35], [36]. In contrast, no mutation in the *TRMU* gene has thus far been identified in Asian populations, such as Chinese or Korean [37], [38], [39]. Our study may therefore provide a novel insight into the pathogenesis of non-syndromic deafness induced by aminoglycoside antibiotics.

## Supporting Information

Figure S1 **Effects of paromomycin and ribostamycin on yeast carrying mitochondrial C1477G mutation and wild type allele.** 10-fold dilutions of each strain were spotted onto YPD or YPD containing indicated antibiotic, and the plates were incubated for 3 days at 30°C.
(TIF)

Table S1 **MIC_90_ values (μg/ml) of neomycin and paromomycin on yeast.**
(DOC)

## References

[1] Ryan MT, Hoogenraad NJ (2007) Mitochondrial-nuclear communications. Annu Rev Biochem 76: 701–722. 17227225 10.1146/annurev.biochem.76.052305.091720

[2] Wallace DC, Fan W, Procaccio V (2010) Mitochondrial energetics and therapeutics. Annu Rev Pathol 5: 297–348. 20078222 10.1146/annurev.pathol.4.110807.092314 PMC3245719

[3] Prezant TR, Agapian JV, Bohlman MC, Bu X, Oztas S, et al (1993) Mitochondrial ribosomal RNA mutation associated with both antibiotic-induced and non-syndromic deafness. Nat Genet 4: 289–294. 7689389 10.1038/ng0793-289

[4] Yuan H, Jiang S, Yang W, Guo W, Cao J, et al (1999) [Screening for mitochondrial 1555(G) mutation in patients with aminoglycoside antibiotic-induced deafness]. Zhonghua Yi Xue Yi Chuan Xue Za Zhi 16: 141–144. 10359861

[5] Estivill X, Govea N, Barcelo E, Badenas C, Romero E, et al (1998) Familial progressive sensorineural deafness is mainly due to the mtDNA A1555G mutation and is enhanced by treatment of aminoglycosides. Am J Hum Genet 62: 27–35. 9490575 10.1086/301676 PMC1376822

[6] Guan MX, Fischel-Ghodsian N, Attardi G (1996) Biochemical evidence for nuclear gene involvement in phenotype of non-syndromic deafness associated with mitochondrial 12S rRNA mutation. Hum Mol Genet 5: 963–971. 8817331 10.1093/hmg/5.7.963

[7] Guan MX, Fischel-Ghodsian N, Attardi G (2001) Nuclear background determines biochemical phenotype in the deafness-associated mitochondrial 12S rRNA mutation. Hum Mol Genet 10: 573–580. 11230176 10.1093/hmg/10.6.573

[8] Yan Q, Li X, Faye G, Guan MX (2005) Mutations in MTO2 related to tRNA modification impair mitochondrial gene expression and protein synthesis in the presence of a paromomycin resistance mutation in mitochondrial 15 S rRNA. J Biol Chem 280: 29151–29157. 15944150 10.1074/jbc.M504247200 PMC2905382

[9] Li X, Li R, Lin X, Guan MX (2002) Isolation and characterization of the putative nuclear modifier gene MTO1 involved in the pathogenesis of deafness-associated mitochondrial 12 S rRNA A1555G mutation. J Biol Chem 277: 27256–27264. 12011058 10.1074/jbc.M203267200

[10] Ghezzi D, Baruffini E, Haack TB, Invernizzi F, Melchionda L, et al (2012) Mutations of the mitochondrial-tRNA modifier MTO1 cause hypertrophic cardiomyopathy and lactic acidosis. Am J Hum Genet 90: 1079–1087. 22608499 10.1016/j.ajhg.2012.04.011 PMC3370278

[11] Li M, Tzagoloff A, Underbrink-Lyon K, Martin NC (1982) Identification of the paromomycin-resistance mutation in the 15 S rRNA gene of yeast mitochondria. J Biol Chem 257: 5921–5928. 6279619

[12] Kutzleb R, Schweyen RJ, Kaudewitz F (1973) Extrachromosomal inheritance of paromomycin resistance in Saccharomyces cerevisiae. Genetic and biochemical characterization of mutants. Mol Gen Genet 125: 91–98. 4590265 10.1007/BF00292984

[13] Weiss-Brummer B, Huttenhofer A (1989) The paromomycin resistance mutation (parr-454) in the 15 S rRNA gene of the yeast Saccharomyces cerevisiae is involved in ribosomal frameshifting. Mol Gen Genet 217: 362–369. 2671660 10.1007/BF02464905

[14] Qian Y, Guan MX (2009) Interaction of aminoglycosides with human mitochondrial 12S rRNA carrying the deafness-associated mutation. Antimicrob Agents Chemother 53: 4612–4618. 19687236 10.1128/AAC.00965-08 PMC2772318

[15] Zhao H, Li R, Wang Q, Yan Q, Deng JH, et al (2004) Maternally inherited aminoglycoside-induced and nonsyndromic deafness is associated with the novel C1494T mutation in the mitochondrial 12S rRNA gene in a large Chinese family. Am J Hum Genet 74: 139–152. 14681830 10.1086/381133 PMC1181901

[16] Decoster E, Vassal A, Faye G (1993) MSS1, a nuclear-encoded mitochondrial GTPase involved in the expression of COX1 subunit of cytochrome c oxidase. J Mol Biol 232: 79–88. 8392589 10.1006/jmbi.1993.1371

[17] Umeda N, Suzuki T, Yukawa M, Ohya Y, Shindo H, et al (2005) Mitochondria-specific RNA-modifying enzymes responsible for the biosynthesis of the wobble base in mitochondrial tRNAs. Implications for the molecular pathogenesis of human mitochondrial diseases. J Biol Chem 280: 1613–1624. 15509579 10.1074/jbc.M409306200

[18] Colby G, Wu M, Tzagoloff A (1998) MTO1 codes for a mitochondrial protein required for respiration in paromomycin-resistant mutants of Saccharomyces cerevisiae. J Biol Chem 273: 27945–27952. 9774408 10.1074/jbc.273.43.27945

[19] Guan MX, Yan Q, Li X, Bykhovskaya Y, Gallo-Teran J, et al (2006) Mutation in TRMU related to transfer RNA modification modulates the phenotypic expression of the deafness-associated mitochondrial 12S ribosomal RNA mutations. Am J Hum Genet 79: 291–302. 16826519 10.1086/506389 PMC1559489

[20] Fan-Minogue H, Bedwell DM (2008) Eukaryotic ribosomal RNA determinants of aminoglycoside resistance and their role in translational fidelity. RNA 14: 148–157. 18003936 10.1261/rna.805208 PMC2151042

[21] Pfister P, Corti N, Hobbie S, Bruell C, Zarivach R, et al (2005) 23S rRNA base pair 2057–2611 determines ketolide susceptibility and fitness cost of the macrolide resistance mutation 2058A–>G. Proc Natl Acad Sci U S A 102: 5180–5185. 15795375 10.1073/pnas.0501598102 PMC555689

[22] Weiss A, Delproposto J, Giroux CN (2004) High-throughput phenotypic profiling of gene-environment interactions by quantitative growth curve analysis in Saccharomyces cerevisiae. Anal Biochem 327: 23–34. 15033507 10.1016/j.ab.2003.12.020

[23] Raimundo N, Song L, Shutt TE, McKay SE, Cotney J, et al (2012) Mitochondrial stress engages E2F1 apoptotic signaling to cause deafness. Cell 148: 716–726. 22341444 10.1016/j.cell.2011.12.027 PMC3285425

[24] Wang Y, Singh U, Mueller DM (2007) Mitochondrial genome integrity mutations uncouple the yeast Saccharomyces cerevisiae ATP synthase. J Biol Chem 282: 8228–8236. 17244612 10.1074/jbc.M609635200 PMC3670140

[25] Bruckmann A, Hensbergen PJ, Balog CI, Deelder AM, Brandt R, et al (2009) Proteome analysis of aerobically and anaerobically grown Saccharomyces cerevisiae cells. J Proteomics 71: 662–669. 19070690 10.1016/j.jprot.2008.11.012

[26] Wang X, Yan Q, Guan MX (2007) Deletion of the MTO2 gene related to tRNA modification causes a failure in mitochondrial RNA metabolism in the yeast Saccharomyces cerevisiae. FEBS Lett 581: 4228–4234. 17706197 10.1016/j.febslet.2007.07.067

[27] Moore PA, Sagliocco FA, Wood RM, Brown AJ (1991) Yeast glycolytic mRNAs are differentially regulated. Mol Cell Biol 11: 5330–5337. 1922048 10.1128/mcb.11.10.5330-5337.1991 PMC361600

[28] Goncalves P, Planta RJ (1998) Starting up yeast glycolysis. Trends Microbiol 6: 314–319. 9746941 10.1016/s0966-842x(98)01305-5

[29] Ralser M, Wamelink MM, Struys EA, Joppich C, Krobitsch S, et al (2008) A catabolic block does not sufficiently explain how 2-deoxy-D-glucose inhibits cell growth. Proc Natl Acad Sci U S A 105: 17807–17811. 19004802 10.1073/pnas.0803090105 PMC2584745

[30] Fourmy D, Recht MI, Puglisi JD (1998) Binding of neomycin-class aminoglycoside antibiotics to the A-site of 16 S rRNA. J Mol Biol 277: 347–362. 9514735 10.1006/jmbi.1997.1552

[31] Ogle JM, Ramakrishnan V (2005) Structural insights into translational fidelity. Annu Rev Biochem 74: 129–177. 15952884 10.1146/annurev.biochem.74.061903.155440

[32] Feldman MB, Terry DS, Altman RB, Blanchard SC (2010) Aminoglycoside activity observed on single pre-translocation ribosome complexes. Nat Chem Biol 6: 54–62. 19946275 10.1038/nchembio.274 PMC2914512

[33] Sasarman F, Antonicka H, Horvath R, Shoubridge EA (2011) The 2-thiouridylase function of the human MTU1 (TRMU) enzyme is dispensable for mitochondrial translation. Hum Mol Genet 20: 4634–4643. 21890497 10.1093/hmg/ddr397

[34] de Moraes VC, Alexandrino F, Andrade PB, Camara MF, Sartorato EL (2009) Study of modifiers factors associated to mitochondrial mutations in individuals with hearing impairment. Biochem Biophys Res Commun 381: 210–213. 19338775 10.1016/j.bbrc.2009.02.014

[35] Zeharia A, Shaag A, Pappo O, Mager-Heckel AM, Saada A, et al (2009) Acute infantile liver failure due to mutations in the TRMU gene. Am J Hum Genet 85: 401–407. 19732863 10.1016/j.ajhg.2009.08.004 PMC2771591

[36] Schara U, von Kleist-Retzow JC, Lainka E, Gerner P, Pyle A, et al (2011) Acute liver failure with subsequent cirrhosis as the primary manifestation of TRMU mutations. J Inherit Metab Dis 34: 197–201. 21153446 10.1007/s10545-010-9250-z

[37] Lu J, Li Z, Zhu Y, Yang A, Li R, et al (2010) Mitochondrial 12S rRNA variants in 1642 Han Chinese pediatric subjects with aminoglycoside-induced and nonsyndromic hearing loss. Mitochondrion 10: 380–390. 20100600 10.1016/j.mito.2010.01.007 PMC2874659

[38] Young WY, Zhao L, Qian Y, Wang Q, Li N, et al (2005) Extremely low penetrance of hearing loss in four Chinese families with the mitochondrial 12S rRNA A1555G mutation. Biochem Biophys Res Commun 328: 1244–1251. 15708009 10.1016/j.bbrc.2005.01.085

[39] Bae JW, Kim DB, Choi JY, Park HJ, Lee JD, et al (2012) Molecular and clinical characterization of the variable phenotype in Korean families with hearing loss associated with the mitochondrial A1555G mutation. PLoS One 7: e42463. 22879993 10.1371/journal.pone.0042463 PMC3412860

