# Supplementary figures and images for: Nuclear Modifier MTO2 Modulates the Aminoglycoside-Sensitivity of Mitochondrial 15S rRNA C1477G Mutation in Saccharomyces cerevisiae
Source: PLoS One. 2013 Dec 10;8(12):e81490. doi: 10.1371/journal.pone.0081490 (PMC3858254; doi:10.1371/journal.pone.0081490)

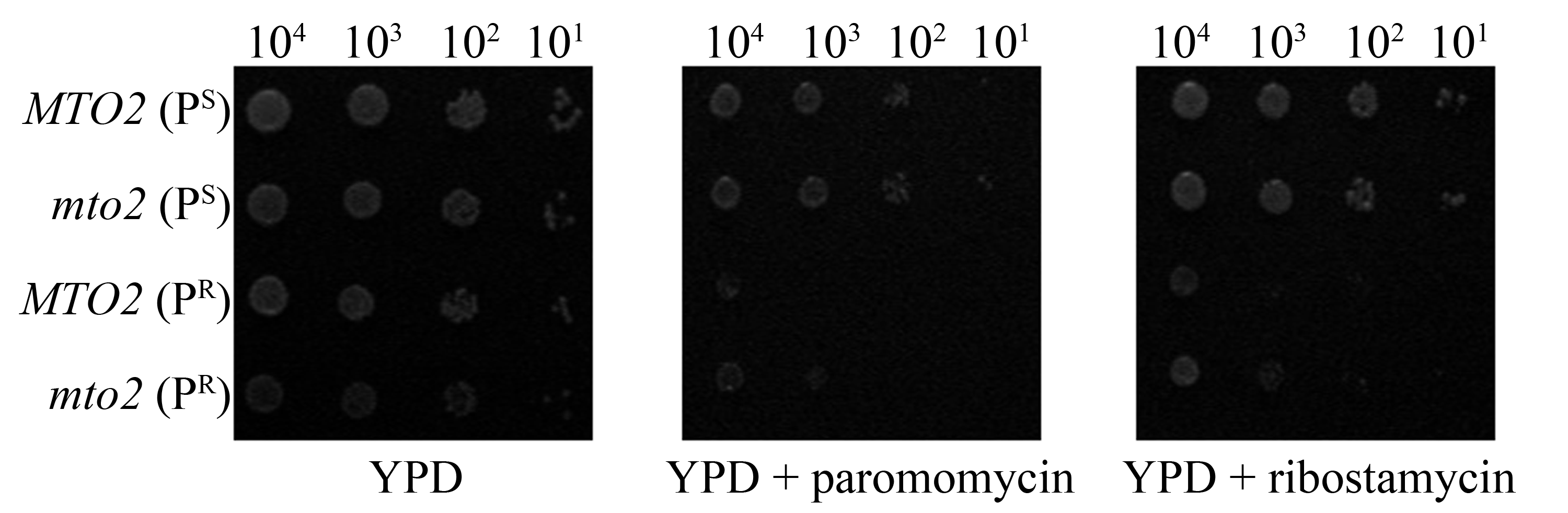

Supplement: Figure S1 — Effects of paromomycin and ribostamycin on yeast carrying mitochondrial C1477G mutation and wild type allele. 10-fold dilutions of each strain were spotted onto YPD or YPD containing indicated antibiotic, and the plates were incubated for 3 days at 30°C. (TIF) [file pone.0081490.s001.tif]
