# Supplementary material for: Nuclear Modifier MTO2 Modulates the Aminoglycoside-Sensitivity of Mitochondrial 15S rRNA C1477G Mutation in Saccharomyces cerevisiae
Source: PLoS One. 2013 Dec 10;8(12):e81490. doi: 10.1371/journal.pone.0081490 (PMC3858254; doi:10.1371/journal.pone.0081490)
Supplement: Table S1 — MIC90 values (μg/ml) of neomycin and paromomycin on yeast. (DOC) [file pone.0081490.s002.doc]

**Table S1. MIC_90_ values (μg/ml) of neomycin and paromomycin on yeast**

| Genotypes  Antibiotics | *MTO2*(P^S^) | *mto2*(P^S^) | *MTO2*(P^R^) | *mto2*(P^R^) |
| --- | --- | --- | --- | --- |
| Neomycin | 128 | 128 | 32 | 64 |
| Paromomycin | 4096 | 4096 | 256 | 512 |
